# Structure Based Protein Engineering of Aldehyde Dehydrogenase from *Azospirillum brasilense* to Enhance Enzyme Activity against Unnatural 3-Hydroxypropionaldehyde

**DOI:** 10.4014/jmb.2110.10038

**Published:** 2021-12-04

**Authors:** Hyeoncheol Francis Son, Kyung-Jin Kim

**Affiliations:** 1 KNU Institute for Microorganisms, Kyungpook National University, Daegu 41566, Republic of Korea; 2 School of Life Sciences, BK21 FOUR KNU Creative BioResearch Group, Kyungpook National University, Daegu 41566, Republic of Korea

**Keywords:** 3-Hydroxypropionate, aldehyde dehydrogenase, *Azospirillum brasilense*

## Abstract

3-Hydroxypropionic acid (3HP) is a platform chemical and can be converted into other valuable C3-based chemicals. Because a large amount of glycerol is produced as a by-product in the biodiesel industry, glycerol is an attractive carbon source in the biological production of 3HP. Although eight 3HP-producing aldehyde dehydrogenases (ALDHs) have been reported so far, the low conversion rate from 3-hydroxypropionaldehyde (3HPA) to 3HP using these enzymes is still a bottleneck for the production of 3HP. In this study, we elucidated the substrate binding modes of the eight 3HP-producing ALDHs through bioinformatic and structural analysis of these enzymes and selected protein engineering targets for developing enzymes with enhanced enzymatic activity against 3HPA. Among ten *Ab*KGSADH variants we tested, three variants with replacement at the Arg281 site of *Ab*KGSADH showed enhanced enzymatic activities. In particular, the *Ab*KGSADH^R281Y^ variant exhibited improved catalytic efficiency by 2.5-fold compared with the wild type.

## Introduction

3HP is one of the top building block chemicals issued in 2004 and 2010 by the United States Department of Energy. As a platform chemical, 3HP can be converted into other valuable C3-based chemicals, such as acrylic acid, acrylamide, malonic acid, and other 3HP- or acryl-based polymers [1-4]. Several chemical approaches to produce 3HP industrially using oxidation from 1,3-propanediol or 3HPA, and hydration from acrylic acid have been reported [5, 6]. However, several advantages associated with free of petroleum-based raw materials, low cost, and environmental issues are driving the production of 3HP through biological methods [7].

For the biological production of 3HP, two biosynthetic pathways have been developed using glucose or glycerol as carbon sources. In the glucose pathway, 3HP can be produced by a carbon fixation pathway such as the 3HP/ 4HB cycle [8-11]. In the glycerol pathway, glycerol dehydratase converts glycerol to 3HPA, and then ALDH converts 3HPA to 3HP [12, 13]. In recent years, a large amount of glycerol has been produced as a byproduct in the biodiesel industry, therefore glycerol is attracting attention as a suitable carbon source for 3HP production [14, 15].

Eight 3HP-producing ALDH enzymes have been reported to date, including *Bs*DhaS, *Cn*GapD4, *Ec*AldH, *Kp*PuuC, *Kp*YdcW, *Kp*YneI, *Sc*Ald4, and *Ab*KGSADH (Table S1) [16-22]. Among these, only one crystal structure of *Ab*KGSADH has been reported, and this report explained how *Ab*KGSADH stabilize to catalyze various chemicals such as α-ketoglutaric semialdehyde, succinic semialdehyde, and 3-HPA [23]. The structural information was also utilized to enhance its reactivity [24]. However, enzyme reactivity of ALDH for the conversion of 3HPA to 3HP is still low, and the activity imbalance in between DhaB and ALDH causes toxic 3HPA to accumulate in the cell. Moreover, the high expression of ALDH enzyme can be a burden on the host strain, therefore ALDH increasing activity by enhancing 3HPA specificity has been required [25].

Here, we elucidate the unique substrate specificity of ALDH enzymes by bioinformatic analysis. Crystal structure of *Ab*KGSADH, structure homology modeling, and molecular docking simulation revealed unnatural 3HPA binding modes of eight 3HP-producing ALDHs, and various amino acid residues are positioned in each substrate binding pocket of ALDHs. Based on this information, ten *Ab*KGSADH variants were constructed and several variants with enhanced activity were obtained successfully. The results from this study could be utilized for the improved production of 3HP.

## Materials and Method

### Protein Homology Modeling

Protein homology modeling was performed using SWISS-MODEL [26, 27]. Model structures of seven 3HP-producing ALDHs were built. NCBI accession codes of target sequences are: AFQ57871.1 (*Bs*DhaS), WP_011617618.1 (*Cn*GapD4), OSL13388.1 (*Ec*AldH), WP_020323708.1 (*Kp*PuuC), ABR77355.1 (*Kp*YdcW), WP_015958390.1 (*Kp*YneI), and NP_015019.1 (*Sc*Ald4). PDB codes of template structures, amino acid sequence identity between query and template sequences, and QMEAN scores are shown in Table S2.

### Molecular Docking Simulation

Molecular docking simulations of 3HPA to 3HP-producing ALDH structures were performed by AutoDock Vina software [28]. Crystal structures of *Ab*KGSADH (PDB code 5X5U) [23] and seven model structures of 3HP-producing ALDHs, including *Bs*DhaS, *Cn*GapD4, *Ec*AldH, *Kp*PuuC, *Kp*YdcW, *Kp*YneI, and *Sc*Ald4, were superimposed and then docking simulations were performed. The 3HPA ligand structure was prepared using the JLigand software [29]. The *pdbqt* files were generated using AutoDock Tools, and all steps were performed by the AutoDock Vina manual [30]. The grid size for *Ab*KGSADH was x = 44, y = 40, z = 34, and the grid center was designated at x = -31.496, y = -11.696, z = -2.0. The grid size for *Bs*DhaS was x = 44, y = 40, z = 32, and the grid center was designated at x = -31.725, y = -11.576, z = -2.897. The grid size for *Cn*GapD4 was x = 44, y = 40, z = 32, and the grid center was designated at x = -31.227, y = -11.339, z = -2.604. The grid size for *Ec*AldH was x = 42, y = 40, z = 32, and the grid center was designated at x = -31.382, y = -11.676, z = -3.182. The grid size for *Kp*PuuC was x = 44, y = 40, z = 40, and the grid center was designated at x = -31.571, y = -11.640, z = -2.65. The grid size for *Kp*YdcW was x = 44, y = 40, z = 32, and the grid center was designated at x = -30.644, y = -10.669, z = -3.018. The grid size for *Kp*YneI was x = 42, y = 40, z = 42, and the grid center was designated at x = -30.794, y = -11.876, z = -2.618. The grid size for *Sc*Ald4 was x = 44, y = 40, z = 32, and the grid center was designated at x = -31.586, y = -11.833, z = -2.735. 90 docking poses in each ALDH were generated, and one pose with the most appropriate direction and distance in between aldehyde group of 3HPA and catalytic residue was selected

### Site-Directed Mutagenesis and Enzyme Preparation

Site-directed mutations were performed using a QuikChange kit (Agilent, USA), and mutated nucleotide sequences were confirmed. *Ab*KGSADH enzyme was prepared with same method as described in our previous study [23]. *Ab*KGSADH wild type and variants sub-cloned into pProEX-HTa vector (Thermo Fischer Scientific, USA) were transformed into an *E. coli* BL21(DE3)-T1^R^ strain, and each strain was cultured to an OD_600_ of 0.6 in fresh LB medium with 100 mg l^-1^ ampicillin at 37°C. *Ab*KGSADH protein expression was induced by 0.5 mM IPTG. After 20 h at 18°C, the cells were harvested by 4,000 g for 15 min at 4°C. The cells were resuspended in ice-cold 40 mM Tris-HCl, pH 8.0, and disrupted by ultrasonication. Cell debris was removed by centrifugation at 13,000 g for 30 min, and the lysate was applied onto a Ni-NTA agarose column (Qiagen, Germany). After washing with 40 mM Tris and 25 mM imidazole, pH 8.0, the *Ab*KGSADH protein was eluted with 40 mM Tris and 300 mM imidazole, pH 8.0.

### Enzyme Activity Assay

The activity of *Ab*KGSADH wild type and variants was determined by measuring the increase of absorbance at 340 nm. Enzyme reaction was performed with a reaction mixture of 0.5 mM total volume at 25°C. For kinetic analysis, reaction mixtures contained 100 mM Tris-HCl, pH 8.0, 500 μM NAD, 50 nM *Ab*KGSADH enzyme, and various concentrations of 3HPA (0.5-10 mM). The reactions were initiated by the addition of enzyme. All reactions were performed in duplicate. The initial velocity of each measurement was calculated with extinction coefficient of NADH (6.22 M^-1^ cm^-1^). Statistical analysis for K_M_ and *k*_cat_ values was performed by Michaelis Menten models using OriginPro software (OriginLab, USA).

## Results and Discussion

### Unnatural Substrate Availability of ALDHs

According to the enzyme nomenclature databases operated by the Nomenclature Committee of the International Union of Biochemistry and Molecular Biology, ALDHs belonging to the EC 1.2.1.- group (oxidoreductases acting on the aldehyde- or oxo-group of donors with NAD or NADP as acceptor) are divided into 107 classes (1.2.1.1-107) based on their specific activity [31]. Among these 107 classes, 2 classes have been deleted, 12 classes have been transferred to another entry, and 13 classes have no annotated nucleotide/amino acid sequence data in the database. Another 34 classes have amino acid sequences that refer to their entries, but no crystal structure has been reported to verify their structural fold and elucidate detailed molecular mechanisms and catalyses. Finally, the selected crystal structures of the remaining 46 different ALDH classes were compared (Tables S3, S4). Interestingly, when the selected crystal structures were superimposed on each other, approximately 70% of the ALDH classes displayed a similar overall structure (ALDH fold) and the remaining ALDHs have the GapDH fold (15%) or their own unique shape (15%) (Tables S3, S4). Moreover, many enzymes with the ALDH fold are multi-classified in the EC 1.2.1.- category. These observations indicate that most ALDH enzymes have similar structural conformations, and differences in only several amino acids involved in the constitution of the substrate binding pocket cause ALDH enzymes to accommodate different substrates. Due to these structural properties, ALDH enzymes have relatively broad substrate specificities, which in turn allow some ALDH enzymes to utilize unnatural aldehyde chemicals as substrates.

### Unnatural 3HPA Binding Mode Prediction

The oxidation reaction from 3HPA to 3HP is a key step in the efficient production of 3HP from glycerol. However, because enzymes utilizing the unnatural 3HPA as a substrate have not yet been reported in natural organisms, use of the unique enzyme property of the ALDH enzymes with a broad substrate specificity is crucial for the enzymatic conversion of 3HPA to 3HP. Although eight 3HP-producing ALDHs have been reported to date, their low activities against 3HPA still remain a bottleneck for highly efficient 3HP production [16-22]. Here, an understanding of the substrate binding sites of the eight ALDHs and detailed structural comparisons of these ALDHs can be considered essential for the development of the ALDH enzymes with high activity against 3HPA. Because the crystal structure was reported only in *Ab*KGSADH among the eight 3HP-producing ALDHs [23], we first attempted to obtain the three-dimensional structure of the seven other ALDHs through homology modeling. We used the known structure with the highest amino acid homology to each ALDH as a template model for more accurate structural prediction, and all seven modeled structures had reasonable model quality estimation scores (Table S2).

**Fig. 1:**
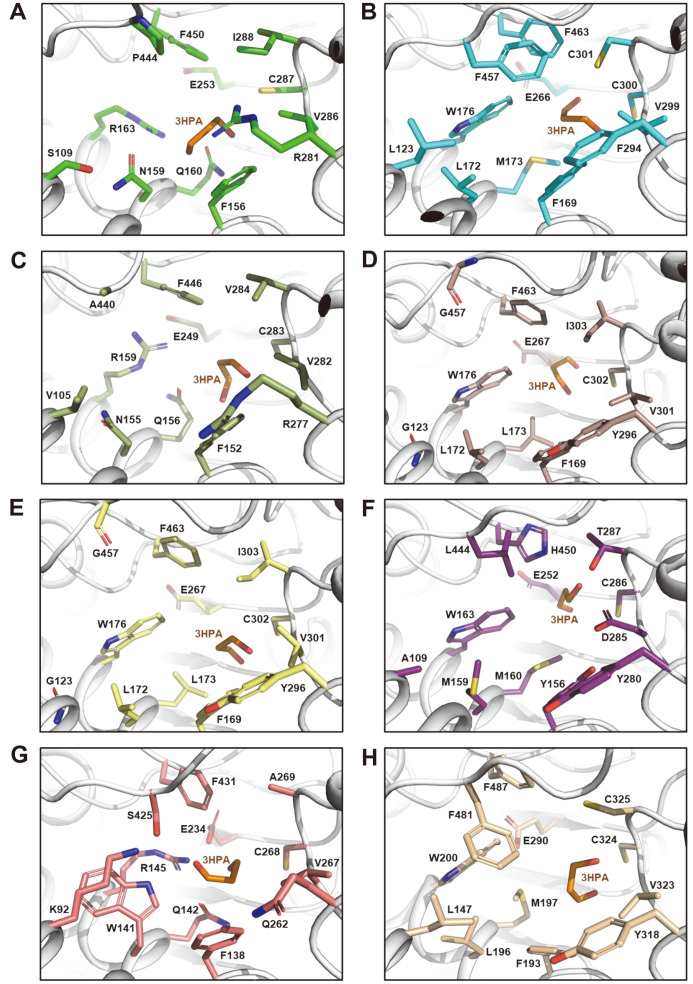
Homology modelling and docking simulation of 3HP-producing ALDHs. (**A-H**) Molecular docking simulation of 3HPA into the *Ab*KGSADH structure (**A**) and *Bs*DhaS (**B**), *Cn*GapD4 (**C**), *Ec*AldH (**D**), *Kp*PuuC (**E**), *Kp*YdcW (**F**), *Kp*YneI (**G**), and *Sc*Ald4 (**H**) models. ALDH molecules and the 3HPA ligand are shown as a gray-colored cartoon diagram and an orange-colored stick model, respectively. The core residues in the eight 3HP-producing ALDHs are distinguished by various colors, and each residue is labeled appropriately.

**Fig. 2:**
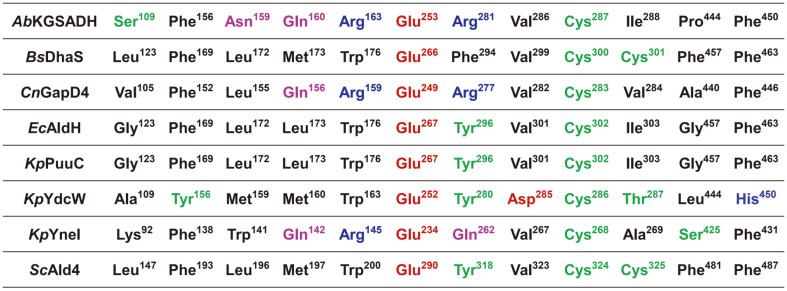
Sequence alignment of core residues contributing 3HPA binding pocket formation. Twelve amino acid residues contributing unnatural 3HPA substrate binding pocket are aligned. Graphical position weight matrix at the top of the alignment table shows the dominance of each site amino acid.

We also performed the molecular docking simulations of the 3HPA molecule into the eight 3HP-producing ALDHs to identify the 3HPA binding mode of these enzymes (Fig. 1). Although the 3HPA molecule was positioned in a slightly different mode in each enzyme, the aldehyde group where the catalysis occurs was directed toward the catalytic residues in all eight enzymes. When we compared the structures of the eight 3HP-producing ALDHs, the two catalytic residues, Glu253 and Cys287 in *Ab*KGSADH, were completely conserved in all eight enzymes, indicating that these enzymes catalyze the reaction in an identical manner (Fig. 1). We then selected ten residues located in the vicinity of the 3HPA molecule and compared these residues in eight 3HP-producing ALDHs. Among eight enzymes, the *Ec*AldH and *Kp*PuuC enzymes, with 83% sequence identity, contained all ten residues, and the other six enzymes contained variable residues (Fig. 2). Of the ten selected residues, three residues, Phe156, Val286, and Phe450 in *Ab*KGSADH, were highly conserved in the 3HP-producing ALDHs, with the exception of *Kp*YneI, which contained Tyr156, Asp285, and His450 residues, respectively. Seven other residues, including Ser109, Asn159, Gln160, Arg163, Arg281, Ile288, and Pro444 in *Ab*KGSADH, were variable in the 3HP-producing ALDHs (Fig. 2). In particular, three residues, Ser109, Ile288, and Pro444 in *Ab*KGSADH, were highly variable, so that a dominant residue was not found (Fig. 2).

### Protein Engineering of *Ab*KGSADH to Improve 3HPA Utilization

As we described above, the residues involved in stabilization of the 3HPA substrate are quite diverse among the known 3HP-producing ALDHs, and these variable residues can be potential targets for enzyme engineering for more efficient 3HP production. Of ten residues involved in substrate binding, we selected four relatively more variable residues, Ser109, Arg281, Ile288, and Pro444 in *Ab*KGSADH, for target engineering sites. We then replaced these four residues of *Ab*KGSADH with the various corresponding residues located in seven other 3PH-producing ALDHs, and generated the following ten *Ab*KGSADH variants: *Ab*KGSADH^S109G^, *Ab*KGSADH^S109L^, *Ab*KGSADH^R281Y^, *Ab*KGSADH^R281F^, *Ab*KGSADH^R281Q^, *Ab*KGSADH^I288T^, *Ab*KGSADH^P444F^, *Ab*KGSADH^P444G^, *Ab*KGSADH^P444L^, and *Ab*KGSADH^P444S^.

**Fig. 3:**
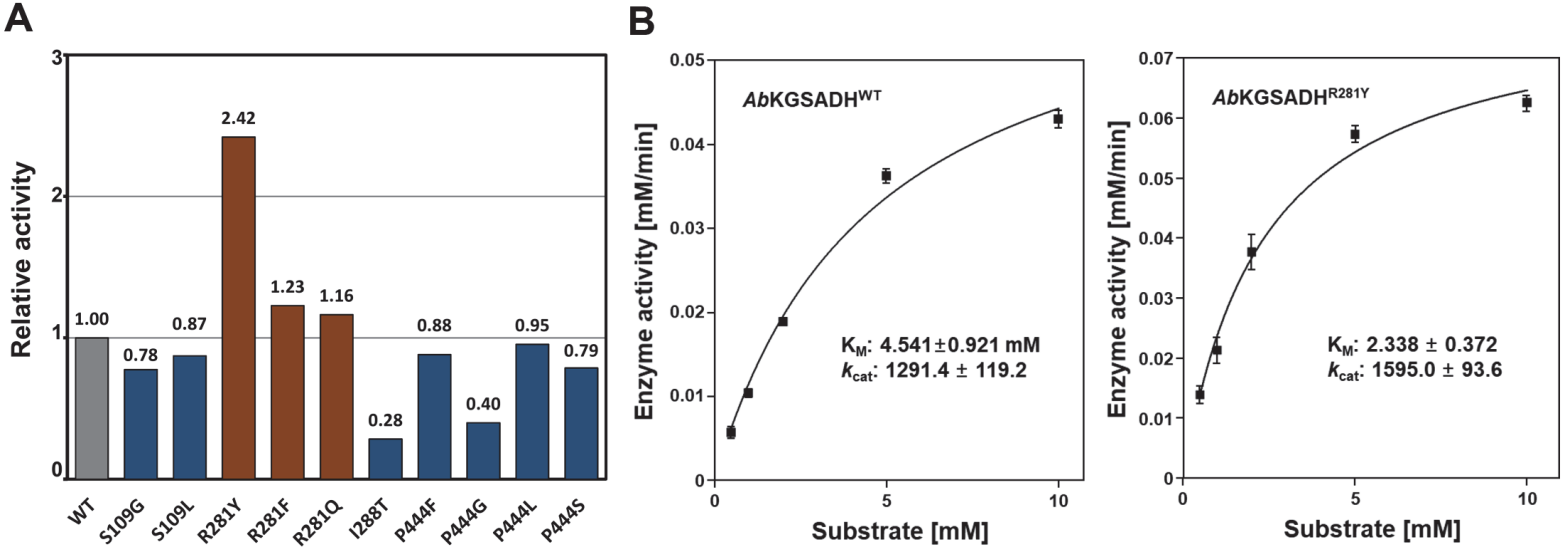
Kinetic parameters and relative catalytic efficiency. (**A**) Relative catalytic efficiency represented by *k*_cat_/K_M_ value. The catalytic efficiency of ten variants were compared with that of wild type *Ab*KGSADH. (**B**) Enzyme kinetic graphs of *Ab*KGSADHWT (left) and *Ab*KGSADH^R281Y^ (right).

We measured the specific activities and kinetic parameters of these ten variants and compared them with the same parameters for *Ab*KGSADH^WT^. The *Ab*KGSADH^S109G^ and *Ab*KGSADH^S109L^ variants showed somewhat decreased activities compared with *Ab*KGSADH^WT^ (Fig. 3A). The kinetic parameters of these two variants indicate that the decreased activities are due to decreased *k*_cat_ values rather than decreased substrate affinity (Table 1). The phenomenon was more dramatic in the *Ab*KGSADH^S109G^ variant, and we suspect that replacement of Ser109 by a small hydrophobic glycine increased the binding affinity for 3HPA, however, the altered binding conformation to the molecule by the replacement severely hampered the conversion ability of the variant. The *Ab*KGSADH^I288T^ variant showed only 28% activity compared to the wild type, and kinetic analysis of the variant revealed that decreased activity was due mainly to decreased *k*_cat_ value (Fig. 3A and Table 1). In the case of the variants mutated at the Pro444 site, all four variants showed decreased activities compared with the wild type (Fig. 3A). Kinetic analysis of the four variants indicated that the levels of decreased activities were almost proportional to the levels of decreased *k*_cat_ values, with the K_M_ values similar to each other (Table 1). Based on these observations, we conclude that three residues, Ser109, Ile288, and Pro444 of *Ab*KGSADH, provide a more suitable conformation for 3HP binding and enzyme catalysis compared with the residues possessed by other 3HP-producing enzymes.

Interestingly, the three variants mutated at the Arg281 position of *Ab*KGSADH exhibited substantially increased catalytic efficiencies. The *Ab*KGSADH^R281F^ variant displayed a *k*_cat_ value almost identical to *Ab*KGSADH^WT^, but displayed a dramatically decreased K_M_ value, resulting in 23% enhanced catalytic efficiency for this variant. The *Ab*KGSADH^R281Q^ variant displayed higher values in both *k*_cat_ and K_M_, resulting in a 16%improvement in catalytic efficiency for this variant. The most remarkable improvement was observed in the *Ab*KGSADH^R281Y^ variant. This variant showed 2.5-fold increased catalytic efficiency with approximately half the K_M_ value and 24% higher *k*_cat_ value compared with *Ab*KGSADH^WT^, indicating that the enhanced catalytic efficiency was due to both the higher substrate affinity and increased conversion rate to product (Table 1 and Figs. 3A and 3B). Based on these results, we believe that the location of an amino acid with a bulky or ring side-chain, such as Arg, Gln, Phe, and Tyr, at the position of Arg281 of *Ab*KGSADH is crucial for stabilization of the 3HPA substrate. In particular, the tyrosine residue appears to be most optimal for the unnatural 3HPA substrate, and in fact, half of the 3HP-producing ALDHs have the tyrosine residue at this site.

In summary, bioinformatic analysis of ADLHs belonging to the EC 1.2.1.- category shows that most ALDHs have the same overall fold, and their substrate specificity depends on amino acid residues involved in the formation of the substrate binding pocket. ALDH enzymes with a conventional ALDH fold can utilize a wide range of aldehyde chemicals as substrates, indicating that unnatural aldehydes can also be catalyzed by these ALDHs. Structure homology modeling and molecular docking simulation allowed us to identify detailed substrate binding modes of the eight 3HP-producing ALDHs reported to date and determine amino acids that can be protein engineering targets to improve utilization of the unnatural 3HPA substrate. Of the ten *Ab*KGSADH variants, three variants in which the Arg281 site of *Ab*KGSADH was mutated showed enhanced 3HPA utilization ability. In particular, *Ab*KGSADH^R281Y^ exhibited improved catalytic efficiency by 2.5-fold. This study offers valuable information in the pursuit of highly efficient 3HP production.

## Supplemental Materials

Supplementary data for this paper are available on-line only at http://jmb.or.kr.

## Figures and Tables

**Table 1:** Kinetic parameters of *Ab*KGSADH wild type and ten variants.

*Ab*KGSADH	*k*_cat_ [min^-1^]	K_M_ [mM]	*k*_cat_/K_M_ [(mM min)^-1^]
Wild type	1291.4 ± 119.2	4.581 ± 0.921	281.9
S109G	359.8 ± 52.6	1.646 ± 0.726	218.5
S109L	1021.8 ± 149.8	4.156 ± 1.377	245.9
R281Y	1595.0 ± 93.6	2.338 ± 0.372	682.3
R281F	1317.0 ± 108.2	3.808 ± 0.728	345.9
R281Q	1891.6 ± 236.6	5.764 ± 1.462	328.2
I288T	367.0 ± 101.2	4.593 ± 2.814	79.9
P444F	1171.0 ± 62.6	4.714 ± 0.547	248.4
P444G	395.2 ± 95.0	3.512 ± 2.070	112.5
P444L	1130.4 ± 141.4	4.204 ± 1.184	268.9
P444S	1039.8 ± 305.8	4.690 ± 2.996	221.7

## References

[1] Borodina I, Kildegaard KR, Jensen NB, Blicher TH, Maury J, Sherstyk S (2015). Establishing a synthetic pathway for high-level production of 3-hydroxypropionic acid in *Saccharomyces cerevisiae* via beta-alanine. Metab. Eng..

[2] Karp EM, Eaton TR, Nogue VSI, Vorotnikov V, Biddy MJ, Tan ECD (2017). Renewable acrylonitrile production. Science.

[3] Kumar V, Ashok S, Park S (2013). Recent advances in biological production of 3-hydroxypropionic acid. Biotechnol. Adv..

[4] Valdehuesa KNG, Liu HW, Nisola GM, Chung WJ, Lee SH, Park SJ (2013). Recent advances in the metabolic engineering of microorganisms for the production of 3-hydroxypropionic acid as C3 platform chemical. Appl. Microbiol. Biotechnol..

[5] Haas T, Brossmer C, Meier M, Arntz D, Freund A (2000). Process for preparing 3-hydroxypropionic acid or its salt. Patent Application No. EP0819670.

[6] Behr A, Botulinski A, Carduck Fj SM (1996). process for preparing 3-hydroxypropionic acid. Patent Application No. EP0579617.

[7] Jiang X, Meng X, Xian M (2009). Biosynthetic pathways for 3-hydroxypropionic acid production. Appl. Microbiol. Biotechnol..

[8] Rathnasingh C, Raj SM, Lee Y, Catherine C, Ashoka S, Park S (2012). Production of 3-hydroxypropionic acid via malonyl-CoA pathway using recombinant *Escherichia coli* strains. J. Biotechnol..

[9] Suyama A, Higuchi Y, Urushihara M, Maeda Y, Takegawa K (2017). Production of 3-hydroxypropionic acid via the malonyl-CoA pathway using recombinant fission yeast strains. J. Biosci. Bioeng..

[10] Hugler M, Huber H, Stetter KO, Fuchs G (2003). Autotrophic CO_2_ fixation pathways in archaea (*Crenarchaeota*). Arch Microbiol..

[11] Hugler M, Menendez C, Schagger H, Fuchs G (2002). Malonyl-coenzyme A reductase from *Chloroflexus aurantiacus*, a key enzyme of the 3-hydroxypropionate cycle for autotrophic CO_2_ fixation. J. Bacteriol..

[12] Forage RG, Foster MA (1982). Glycerol fermentation in *Klebsiella pneumoniae*: functions of the coenzyme B12-dependent glycerol and diol dehydratases. J. Bacteriol..

[13] Ashok S, Raj SM, Rathnasingh C, Park S (2011). Development of recombinant *Klebsiella pneumoniae* Delta dhaT strain for the coproduction of 3-hydroxypropionic acid and 1,3-propanediol from glycerol. Appl. Microbiol. Biotechnol..

[14] Nitayavardhana S, Khanal SK (2011). Biodiesel-derived crude glycerol bioconversion to animal feed: a sustainable option for a biodiesel refinery. Bioresour. Technol..

[15] da Silva GP, Mack M, Contiero J (2009). Glycerol: a promising and abundant carbon source for industrial microbiology. Biotechnol. Adv..

[16] Su M, Li Y, Ge X, Tian P (2015). 3-Hydroxypropionaldehyde-specific aldehyde dehydrogenase from *Bacillus subtilis* catalyzes 3-hydroxypropionic acid production in *Klebsiella pneumoniae*. Biotechnol. Lett..

[17] Chu HS, Kim YS, Lee CM, Lee JH, Jung WS, Ahn JH (2015). Metabolic engineering of 3-hydroxypropionic acid biosynthesis in *Escherichia coli*. Biotechnol. Bioeng..

[18] Jo JE, Raj SM, Rathnasingh C, Selvakumar E, Jung WC, Park S (2008). Cloning, expression, and characterization of an aldehyde dehydrogenase from *Escherichia coli* K-12 that utilizes 3-Hydroxypropionaldehyde as a substrate. Appl. Microbiol. Biotechnol..

[19] Raj SM, Rathnasingh C, Jung WC, Selvakumar E, Park S (2010). A Novel NAD^+^-dependent aldehyde dehydrogenase encoded by the puuC gene of *Klebsiella pneumoniae* DSM 2026 that utilizes 3-hydroxypropionaldehyde as a substrate. Biotechnol. Bioproc. E..

[20] Luo LH, Seo JW, Heo SY, Oh BR, Kim DH, Kim CH (2013). Identification and characterization of *Klebsiella pneumoniae* aldehyde dehydrogenases increasing production of 3-hydroxypropionic acid from glycerol. Bioprocess Biosyst. Eng..

[21] Li Y, Su M, Ge X, Tian P (2013). Enhanced aldehyde dehydrogenase activity by regenerating NAD^+^ in *Klebsiella pneumoniae* and implications for the glycerol dissimilation pathways. Biotechnol. Lett..

[22] Ko Y, Ashok S, Zhou S, Kumar V, Park S (2012). Aldehyde dehydrogenase activity is important to the production of 3-hydroxypropionic acid from glycerol by recombinant *Klebsiella pneumoniae*. Process Biochem..

[23] Son HF, Park S, Yoo TH, Jung GY, Kim KJ (2017). Structural insights into the production of 3-hydroxypropionic acid by aldehyde dehydrogenase from *Azospirillum brasilense*. Sci. Rep. Uk.

[24] Park YS, Choi UJ, Nam NH, Choi SJ, Nasir A, Lee SG (2017). Engineering an aldehyde dehydrogenase toward its substrates, 3-hydroxypropanal and NAD^+^, for enhancing the production of 3-hydroxypropionic acid. Sci. Rep..

[25] Kumar V, Ashok S, Park S (2013). Recent advances in biological production of 3-hydroxypropionic acid. Biotechnol. Adv..

[26] Studer G, Rempfer C, Waterhouse AM, Gumienny R, Haas J, Schwede T (2020). QMEAND is Co-distance constraints applied on model quality estimation. Bioinformatics.

[27] Waterhouse A, Bertoni M, Bienert S, Studer G, Tauriello G, Gumienny R (2018). SWISS-MODEL: homology modelling of protein structures and complexes. Nucleic Acids Res..

[28] Trott O, Olson AJ (2010). AutoDock Vina: improving the speed and accuracy of docking with a new scoring function, efficient optimization, and multithreading. J. Comput. Chem..

[29] Lebedev AA, Young P, Isupov MN, Moroz OV, Vagin AA, Murshudov GN (2012). JLigand: a graphical tool for the CCP4 templaterestraint library. Acta Crystallogr. D, Biol. Crystallogr..

[30] Morris GM, Huey R, Lindstrom W, Sanner MF, Belew RK, Goodsell DS (2009). AutoDock4 and autodockTools4: Automated docking with selective receptor flexibility. J. Comput. Chem..

[31] Moss GP Nomenclature Committee of the International Union of Biochemistry and Molecular Biology (NC-IUBMB).

