# Supplementary material for: Structure Based Protein Engineering of Aldehyde Dehydrogenase from Azospirillum brasilense to Enhance Enzyme Activity against Unnatural 3-Hydroxypropionaldehyde
Source: J Microbiol Biotechnol. 2021 Dec 4;32(2):170–5. doi: 10.4014/jmb.2110.10038 (PMC9628839; doi:10.4014/jmb.2110.10038)
Supplement: Supplementary file 1 [file jmb-32-2-170-supple.pdf]

Supplementary Data

Structure based protein engineering of aldehyde dehydrogenase from *Azospirillum brasilense* to enhance enzyme activity against unnatural 3-hydroxypropionaldehyde

Hyeoncheol Francis Son[1] and Kyung-Jin Kim[1,2*]

[1]KNU Institute for Microorganisms, Kyungpook National University, Daehak-ro 80, Buk-ku, Daegu, 41566, Republic of Korea

[2]School of Life Sciences, BK21 FOUR KNU Creative BioResearch Group, Kyungpook National University, Daegu 41566, Korea

Table S1. Amino acid sequence identity in between 3HP producing ALDHs.

	KGSADH	DhaS	GapD4	AldH	PuuC	YdcW	YneI	Ald4
*Ab*KGSADH	**100**							
*Bs*DhaS	**35.6**	**100**						
*Cn*GapD4	**49.9**	**35.4**	**100**					
*Ec*AldH	**34.1**	**41.3**	**34.5**	**100**				
*Kp*PuuC	**33.9**	**42.7**	**36.9**	**83.0**	**100**			
*Kp*YdcW	**33.1**	**39.3**	**32.8**	**38.1**	**37.7**	**100**		
*Kp*YneI	**33.1**	**29.4**	**31.1**	**33.0**	**33.0**	**29.1**	**100**	
*Sc*Ald4	**31.3**	**49.0**	**34.4**	**37.8**	**38.8**	**36.8**	**32.0**	**100**

Abbreviations: *Ab, Azospirillum brasilense; Bs, Bacillus subtilis; Cn, Cupria3vidus necator; Ec, Escherichia coli; Kp, Klebsiella pneumonia; Sc, Saccharomyces cerevisiae;*

Table S2. Protein homology modeling and molecular docking simulation

	Homology modeling			3HPA docking
	**Template (PDB code)**	**Seq Identity (%)**	**QMEAN**	**Affinity (kcal/mol)**
***Ab*KGSADH**	5X5T*			-3.4
***Bs*DhaS**	5GTK	62.35	0.33	-2.7
***Cn*GapD4**	5VBF	63.71	0.06	-3.5
***Ec*AldH**	5IUV	57.99	-0.41	-2.6
***Kp*PuuC**	5IUV	58.45	-0.20	-2.9
***Kp*YdcW**	6C43	83.97	-0.12	-3.0
***Kp*YneI**	3EFV	74.19	0.12	-3.3
***Sc*Ald4**	5FHZ	50.42	-0.88	-3.5

**Ab*KGSADH crystal structure

Table S2. Enzyme nomenclature of 1.2.1.-

1.2.1.-	Deleted	Transferred	No labeled	Structure			No structure
				ALDH fold	GapDH fold	Others	
107	2	12	13	32	7	7	34
